# Human Campylobacteriosis in Developing Countries1

**DOI:** 10.3201/eid0803.010233

**Published:** 2002-03

**Authors:** Akitoye O. Coker, Raphael D. Isokpehi, Bolaji N. Thomas, Kehinde O. Amisu, C. Larry Obi

**Affiliations:** * University of Lagos, Idi-Araba, Lagos, Nigeria; † University of Venda for Science and Technology, Thohoyandon, South Africa

**Keywords:** *Campylobacter*, *Campylobacter* infections, developing countries, developed countries, epidemiology, public health

## Abstract

Campylobacteriosis is a collective description for infectious diseases caused by members of the bacterial genus *Campylobacter*. The only form of campylobacteriosis of major public health importance is *Campylobacter* enteritis due to *C. jejuni* and *C. coli.* Research and control efforts on the disease have been conducted more often in developed countries than developing countries. However, because of the increasing incidence, expanding spectrum of infections, potential of HIV-related deaths due to *Campylobacter,* and the availability of the complete genome sequence of *C. jejuni* NCTC 11168, interest in campylobacteriosis research and control in developing countries is growing. We present the distinguishing epidemiologic and clinical features of *Campylobacter* enteritis in developing countries relative to developed countries. National surveillance programs and international collaborations are needed to address the substantial gaps in the knowledge about the epidemiology of campylobacteriosis in developing countries.

Campylobacteriosis is a collective description for infectious diseases caused by members of the bacterial genus *Campylobacter*. The only form of campylobacteriosis of major public health importance is *Campylobacter* enteritis due to *C. jejuni* and *C. coli* (*1*). The rate of *Campylobacter* infections worldwide has been increasing, with the number of cases often exceeding those of salmonellosis and shigellosis (*2*). This increase, as well as the expanding spectrum of diseases caused by the organisms, necessitates a clearer understanding of the epidemiology and control of campylobacteriosis.

Surveillance and control of diseases of public health importance in developing countries have focused on diseases such as malaria, tuberculosis, trypanosomiasis, onchocerciasis, and schistosomiasis (*3*). Programs for diarrhea and acute respiratory illness also exist (*4*). These programs have extensive support from the World Health Organization (WHO).

*Campylobacter* is one of the most frequently isolated bacteria from stools of infants with diarrhea in developing countries—a result of contaminated food or water (*5*,*6*). However, national surveillance programs for campylobacteriosis generally do not exist in most developing countries despite the substantial burden of disease. Most data available on campylobacteriosis in developing countries were collected as a result of support provided by WHO to many laboratories in developing countries, including grants for epidemiologic studies and Lior serotyping antisera provided by the Public Health Service of Canada (*5*,*7*). The number of reviews and updates on human campylobacteriosis in developed countries (*8*–*11*) is greater than that for developing countries (*5*,*6*). This disparity may be because *Campylobacter*-associated diarrhea in developing countries is not pathogenic in patients >6 months of age. However, a community-based longitudinal study provided evidence that infection could be pathogenic beyond the first 6 months of life in developing countries (*12*). Furthermore, the sequencing and publication of the complete genome of *C. jejuni* NCTC 11168 have heralded a renaissance of interest in this organism, offering researchers worldwide, including in developing countries, novel ways to contribute to the understanding the organism’s biology (*13*). Thus, to promote research and control of campylobacteriosis in developing countries, review information on human campylobacteriosis in these countries is urgently needed. We present the distinguishing features of campylobacteriosis in developing countries relative to developed countries.

## Incidence

Generally, developing countries do not have national surveillance programs for campylobacteriosis; therefore, incidence values in terms of number of cases for a population do not exist. Availability of national surveillance programs in developed countries has facilitated monitoring of sporadic cases as well as outbreaks of human campylobacteriosis (*2*,*8*–*11*). Most estimates of incidence in developing countries are from laboratory-based surveillance of pathogens responsible for diarrhea. *Campylobacter* isolation rates in developing countries range from 5 to 20% (*6*). Table 1 shows isolation rates for some countries according to WHO regions from studies of diarrhea in children <5 years old (*14*–*25*). Despite the lack of incidence data from national surveys, case-control community-based studies have provided estimates of 40,000 to 60,000/100,000 for children <5 years of age (*6*,*12*). In contrast, the figure for developed countries is 300/100,000 (*8*). Estimates in the general population in developing and developed countries are similar, approximately 90/100,000 (*5*,*6*,*8*), confirming the observation that campylobacteriosis is often a pediatric disease in developing countries. The isolation and incidence rates in some developing countries have increased since their initial reports (*17*). This increase has often been attributed to improved diagnostic methods, but an actual increase in incidence was observed in *Campylobacter*-associated diarrhea in the Caribbean island of Curaçao (*26*).

**Table 1:** Isolation rates of *Campylobacter* from diarrhea specimens from <5-year-olds in selected developing countries

WHO region and country	Isolation rate (%)	Reference
Africa		
Algeria	17.7	14
Cameroon	7.7	15
Ethiopia	13.8	16
Nigeria	16.5	17
Tanzania	18.0	18
Zimbabwe	9.3	19
Americas		
Brazil	9.9	20
Guatemala	12.1	21
Eastern Mediterranean		
Egypt	9.0	12
Jordan	5.5	22
Southeast Asia		
Bangladesh	17.4	23
Thailand	13.0	24
Western Pacific		
Laos	12.1	25

WHO = World Health Organization.

## Age of Infection

In developing countries, *Campylobacter* is the most commonly isolated bacterial pathogen from <2-year-old children with diarrhea (Table 2). The disease does not appear to be important in adults. In contrast, infection occurs in adults and children in developed countries. Poor hygiene and sanitation and the close proximity to animals in developing countries all contribute to easy and frequent acquisition of any enteric pathogen, including *Campylobacter*. Although infections in infants appear to decline with age (Table 2), a comprehensive community-based cohort study in Egypt has shown that infection could be pathogenic regardless the age of the child, underscoring the need for strengthening prevention and control strategies for campylobacteriosis (*12*).

**Table 2:** Age of patients with *Campylobacter* infection in selected developing countries

Countries (ref.)	Age of infection (months)
Nigeria (*17*)	24
Tanzania (*18*)	18
China (*27*)	12-24
Thailand (*28*)	<12 (18.8%) 12-23 (12.3%) 24-59 (10.3%)
Bangladesh (*29*)	≤12 (38.8%) >12 (15.9%)
Egypt (*12*)	0-5 (8%) 6-11 (14%) 12-23 (4%)

## Polymicrobial Infections Involving *Campylobacter*

*Campylobacter* is isolated relatively frequently with another enteric pathogen in patients with diarrhea in developing countries. In some cases half or more patients with *Campylobacter* enteritis also had other enteric pathogens (*23*,*30*). Organisms reported include *Escherichia coli*, *Salmonella*, *Shigella*, *Giardia lamblia*, and *Rotavirus*. Polymicrobial infections involving *Campylobacter* are rare in developed countries (*5**,**6*)*.*

## Isolation of *Campylobacter* in Healthy Children

The recovery of *Campylobacter* organisms from children without diarrhea is common in developing countries. In some reports the isolation rates for symptomatic and asymptomatic children were not statistically significant. Values as high as 14.9% in controls have been observed (*14*). Acquisition of the pathogen because of poor sanitation and contact with animals early in life may explain the isolation from healthy children. *Campylobacter* is not frequently recovered from asymptomatic persons in developed countries, as observed in the Netherlands, where a 0.5% isolation rate has been reported (*9*).

## Seasonal Variation

In developing countries, *Campylobacter* enteritis has no seasonal preference; in contrast, in developed countries epidemics occur in summer and autumn (*2*). Isolation peaks vary from one country to another and also within countries (*12*,*31*,*32*). The lack of seasonal preference may be due to lack of extreme temperature variation as well as lack of adequate surveillance for epidemics (*5*,*6*).

## Distribution of *Campylobacter* Species

*C. jejuni* and *C. coli* are the two main species isolated in developing countries. The isolation rate of *C. jejuni* exceeds that of *C. coli*, similar to observations in most developed countries (*8*,*9*). Lior biotyping and serotyping methods have been used in developing countries to subtype strains of *C. jejuni* and *C. coli* (*5*,*6*). Table 3 shows the distribution of the subtypes from three African countries. Biotype I was the most common, followed by biotype II. The prevalence of specific serotypes only in symptomatic children may indicate virulence traits or treatment, in cases of gastroenteritis (*33*). Furthermore, correlation between biotypes and serotypes isolated from humans and animals indicates that campylobacteriosis is zoonotic (*36*). Penner serotyping scheme and DNA-based typing, extensively used in developed countries, have been proposed for use in developing countries (*37*).

**Table 3:** Distribution of *Campylobacter jejuni* and *C. coli* biotypes and serotypes in three African countries

Countries (ref.)	Biotypes						Serotypes
	*C. jejuni*				*C. coli*		
	I	II	III	IV	I	II	
Nigeria (*33*)	52.5	28.7	-	-	9.9	8.9	1, 8, 11, 20, 28, 45
Central African Republic (*34*)	31.9	11.0	2.4	-	44.0	11.5	-
South Africa (*35*)	95.4	1.5	-	-	3.1		4, 2, 12, 19, 23, 36

Species other than *C.jejuni* and *C. coli—*such as *C. upsaliensis*, *C. concisus*, and aerotolerant campylobacters (*Arcobacter*)—may also be of pathogenic importance; however, diagnostic capacities to determine their distribution are lacking in developing countries (*38*). These other *Campylobacter* species constitute over 50% of campylobacters isolated at the Red Cross Children’s Hospital, Cape Town, South Africa, for example. (A method termed the Cape Town Protocol is used to isolate *Campylobacter* species at this facility [*39*]). This higher incidence is also supported by a 16% isolation rate of *Arcobacter* species in a 4-month survey from poultry drainage water in Lagos, Nigeria (*40*).

## Antibiotic Resistance in *Campylobacter* Isolates

*Campylobacter* enteritis is a self-limiting disease, and antimicrobial therapy is not generally recommended. However, antimicrobial agents are recommended for extraintestinal infections and for treating immunocompromised persons. Erythromycin and ciprofloxacin are drugs of choice (*10*). The rate of resistance to these drugs is increasing in both developed and developing countries, although the incidence is higher in developing countries. Use of these drugs for infections other than gastroenteritis and self-medication are often the causes of resistance in developing countries; in developed countries, resistance is due to their use in food animals and travel to developing countries. The increase in erythromycin resistance in developed countries is often low and stable at approximately 1% to 2%; this is not true for developing countries (*41*,*42*). For example, in 1984, 82% of *Campylobacter* strains from Lagos, Nigeria, were sensitive to erythromycin; 10 years later, only 20.8% were sensitive (*17*). In addition, resistance to another macrolide, azithromycin, was found in 7% to 15% of *Campylobacter* isolates in 1994 and 1995 in Thailand (*43*). The increasing rate of resistance to the fluoroquinolone, ciprofloxacin limits its clinical usefulness. In Thailand, ciprofloxacin resistance among *Campylobacter* species increased from zero before 1991 to 84% in 1995 (*43*). Recent data have shown a marked increase in resistance to quinolones in developed countries (*41*,*42*,*44*–*46*) (Table 4).

**Table 4:** Trends in Resistance to Ciprofloxacin by *Campylobacter jejuni* in selected developed countries up to year 2000

Country	Period	Resistance strains (%)		Ref.
		Initial	Year 2000	
Freiburg, Germany	1992-2000	22	32	41
Styria, Austria	1996-2000	25.2	40.2	42
England and Wales, UK	1993-2000	10	14.8	44
Philadelphia, USA	1995-2000	<10	36	45
Oslo, Norway	1988-2000	6.1	36	46

## *Campylobacter* as a Cause of Travelers’ Diarrhea

Travel to a developing country is a risk factor for acquiring *Campylobacter*-associated diarrhea. The diarrhea is more severe, and strains are associated with antibiotic resistance (*47*,*48*). Furthermore, campylobacteriosis acquired abroad contribute to the number of cases reported in developed countries (*49*). Among Finnish tourists visiting Morocco, the disease was more prevalent in winter months (*50*).

## Clinical Features

The clinical spectrum of *Campylobacter* enteritis ranges from a watery, nonbloody, noninflammatory diarrhea to a severe inflammatory diarrhea with abdominal pain and fever. Disease is less severe in developing countries than in developed countries (*5*,*6*). In developed countries, disease is characterized by bloody stool, fever, and abdominal pain that is often more severe than that observed for *Shigella* and *Salmonella* infections. In developing countries the features reported are watery stool, fever, abdominal pain, vomiting, dehydration, and presence of fecal leukocytes; patients are also often underweight and malnourished (*12**,**31**,**51*)*.* In Lagos, Nigeria, *Campylobacter* enteritis is characterized by a history of watery offensive-smelling stool lasting <5 days (*51*).

## Guillain-Barré Syndrome

Guillain-Barré Syndrome (GBS) is an autoimmune disorder of the peripheral nervous system, which is characterized by acute flaccid paralysis. *C. jejuni* infection is the most frequently identified infection preceding GBS (*52*). In the developing world, sporadic GBS cases associated with *C. jejuni* infection have been reported from Curaçao, China, India, and South Africa (*26*,*53*–*55*). A comparative study between Curaçao and southwest Netherlands indicated that disease in Curaçao was more severe, had a higher incidence of preceding gastroenteritis, and had greater seasonal fluctuation (*26*)*.* Serotype O:19 is most prevalent worldwide, although other serotypes, such as O:1, O:2, O:57, O:16, O:23, O:37, O:41, and O:44, have been reported (*52*). *C. jejuni* strain O:41 appears to be restricted to Cape Town, South Africa, and represents a genetically stable clone (*55*,*56*). Detailed studies of the role of GBS in acute flaccid paralysis in developing countries, especially in polio-endemic areas, are needed.

## *Campylobacter* Infection in the Setting of HIV

*Campylobacter*-associated diarrhea and bacteremia occur in HIV/AIDS patients worldwide. The species isolated include *C. jejuni*, *C. coli*, *C. upsaliensis*, *Arcobacter butzleri, Helicobacter fennelliae*, and *H. cinaedii* (*57*,*58*). The incidence of clinical manifestations is higher than in HIV-negative patients, with substantial mortality and morbidity. Furthermore, antibiotic resistance and recurrent infections have been observed (*59*). The incidence of HIV/AIDS is higher in developing countries than in developed countries and contributes substantially to deaths among <5-year-old children in epidemic settings (*60*). Thus, infants in developing countries are at risk of impaired immunity to *Campylobacter* enteritis. In addition, HIV/AIDS can increase the number of cases of campylobacteriosis in the adult population in these countries. These observations further support the need for improved understanding of the epidemiology of campylobacteriosis in developing countries.

## Immunologic Aspects

In developing countries, such as Bangladesh, Thailand, Central African Republic, and Mexico, healthy children and adults are constantly exposed to *Campylobacter* antigens in the environment. As a consequence, serum antibodies to *Campylobacter* species develop very early in life in children in developing countries, and the levels of such antibodies tend to be much higher than those in children in the developed world such as in the United States (*61*–*64*). In Nigeria, children who had diarrhea and children who were healthy both had antibodies in their sera that could agglutinate *C. jejuni*; the difference in antibody responses between these groups of children was not statistically significant (*65*). Thus, antibody responses alone should be interpreted with caution in diagnosing *Campylobacter* infections.

In spite of shortcomings in the use of antibodies for diagnosis, increase in the level of anti-flagellar antibody had an inverse correlation with the rates of *Campylobacter* enteritis in the Central African Republic (*63*). An age-related relationship in the development of immunity to *Campylobacter* antigens has also been suggested to account for the age-related declines in the case-to-infection ratio and the period of excretion during the convalescent phase (*28*,*66*).

Usually, as age increases, level of antibody tends to increase. At the earliest stages in life (first 6 months), immunoglobulin (Ig) A, IgG, and IgM levels in response to *Campylobacter* infection are minimal, but thereafter increases are observed in response to infection. The poor serologic response during the first 6 months of life may be due either to a primary response to *Campylobacter* or to the presence of maternal antibodies via the placenta or breast milk.

Breast-feeding has been reported to play a role in *C. jejuni-*induced diarrhea. It decreases the number of episodes and the duration of diarrhea (*67*). In Algeria, exclusively breast-fed infants had fewer symptomatic *Campylobacter* infections than infants who were both breast-fed and bottle-fed (*14*).

Results of experimental observations among Mexican children have also shown that immunity to *Campylobacter* after primary infection may prevent bloody diarrhea from developing and subsequently prevent any disease from manifesting (*68*). In the developed world, the epidemiology may be different because most cases are usually primary infections with more severe clinical manifestations, greater numbers of people with bloody diarrhea (50%, as opposed to 15% in developing countries), and a more prolonged duration of excretion (approximately 15 days, compared with 7 days in developing countries) (*28*). The widespread immunity seen among adults in developing countries is absent in adults in developed countries (*64*).

## Sources of Human Campylobacteriosis

*Campylobacter* infection is hyperendemic in developing countries. The major sources of human infections are environmental contamination and foods. Human-to-human transmission as a result of prolonged convalescent-phase excretion and high population density have also been suggested (*5*,*12*), although observations from developed countries show these are less likely factors (*2*).

## Environmental Contamination

Wild birds as well as domestic and companion animals are known reservoirs for *Campylobacter* species, and shedding of the bacteria from them causes contamination of the environment. *C. jejuni* and *C. coli* have been isolated from chickens, goats, sheep, and pigs in developing countries (*69*,*70*). Strains isolated from human and chickens were phenotypically and genotypically correlated, confirming that chickens are an important source of human campylobacteriosis in developing countries (*36*). Poultry is also an important source of campylobacteriosis in developed countries. Extensive epidemiologic investigations have been done in those countries to identify sources of contamination and routes of transmission to humans to facilitate control efforts (*71*). Risk factors for acquiring campylobacters in developing countries include presence of an animal in the cooking area, uncovered garbage in cooking areas, and lack of piped water (*12*).

## Foods

*Campylobacter*-contaminated foods—the result of poor sanitation—are an important potential source of infection in humans. For example, campylobacters were isolated from 40% and 77% of retail poultry meat sold in Bangkok, Thailand, and Nairobi, Kenya, respectively (*72*,*73*). The serotypes of the organisms isolated in Thailand were similar to those of organisms isolated from humans. In Mexico City, a survey of ready-to-eat roasted chickens showed that they were contaminated with campylobacters (*74*). In developed countries, risk factors associated with foods include occupational exposure to farm animals, consumption of raw milk or milk products, and unhygienic food preparation practices (*2*).

## Estimates of Impact of Human Campylobacteriosis in Developing Countries

The Disability Adjusted Life Year (DALY) is the basic unit used in Burden of Disease (BoD) methodology to quantify the impact of disease on a population (*75*). DALYs have been applied in the Dutch population to measure the mean health burden of *Campylobacter*-associated illness in the period 1990–1995 (*76*). The mean estimate was 1,400 DALYs per year; the main determinants of health burden were acute gastroenteritis (440 DALYs), gastroenteritis-related mortality (310 DALYs), and residual symptoms of GBS (340 DALYs). Although data on DALYs due to campylobacteriosis in developing countries are not available, diarrhea, which is a clinical manifestation of campylobacteriosis, was one of the top three causes of death and disease in developing countries in 1990 (*75*). The disease is projected globally to remain one of the top 10 by 2020. (The burden of campylobacteriosis in developing countries may increase by 2020 because HIV is projected to move up to the 10th position from 28th by 2020.) Considering the higher incidence of campylobacteriosis in developing countries, DALYs for the disease in developing countries will likely be higher than those of the Dutch population.

## Conclusions

The incidence of human campylobacteriosis is increasing worldwide and has attracted the attention of WHO (http://www1.oecd.org/agr/prog/sum-copenhagen00.htm). Ssubstantial gaps in knowledge about the epidemiology of campylobacteriosis in developing countries still exist. Present reported estimates of incidence are based on isolation rates from laboratory- and community-based studies conducted from 1980 to 1995. When various socioeconomic and health changes in developing countries are taken into account, these values may have changed considerably. Thus, public health awareness about the problem is needed, as are strengthened diagnostic facilities for campylobacteriosis, with a view towards setting up national surveillance programs. Such programs would determine the incidence rates, epidemiologic risk factors, interaction of HIV/AIDS and campylobacteriosis, seasonal variation, current state of resistance to antimicrobial agents, role of species other than *C. jejuni* and *C. coli,* and the role of campylobacteriosis in GBS. Collaboration among researchers in developed and developing countries needs to be strengthened, leading to development of regional centers of excellence. Funding organizations should provide incentives for North-South collaborations in *Campylobacter* research, as is done in other diseases such as malaria and trypanosomiasis that are endemic in some developing countries. All these should contribute to understanding of the global epidemiology of human campylobacteriosis.
